# Cardiometabolic healthcare for men with prostate cancer: an MD Anderson Cancer Center experience

**DOI:** 10.1186/s40959-023-00186-x

**Published:** 2023-09-13

**Authors:** Andrew W. Hahn, Whittney Thoman, Efstratios Koutroumpakis, Amer Abdulla, Sumit K. Subudhi, Ana Aparicio, Karen Basen-Enngquist, Christopher J. Logothetis, Susan C. Gilchrist

**Affiliations:** 1 Department of Genitourinary Medical Oncology, Division of Cancer Medicine, University of Texas MD Anderson Cancer Center, 1515 Holcombe Blvd. Unit 1374, Houston, TX 77030 USA; 2 Department of Cancer Survivorship, University of Texas MD Anderson Cancer Center, Houston, TX USA; 3 Department of Cardiology, Division of Internal Medicine, University of Texas MD Anderson Cancer Center, Houston, TX USA; 4 Division of Cardiovascular Medicine, Department of Internal Medicine, University of Texas Medical Branch, Galveston, TX USA; 5 Department of Behavioral Science, Division of Cancer Prevention and Population Sciences, University of Texas MD Anderson Cancer Center, Houston, TX USA; 6 Department of Clinical Cancer Prevention, Division of Cancer Prevention and Population Sciences, University of Texas MD Anderson Cancer Center, Houston, TX USA

**Keywords:** Cardiometabolic, Prostate cancer, Cardiovascular, ASCVD, ADT

## Abstract

**Background:**

Men diagnosed with prostate cancer are at risk for competing morbidity and mortality due to cardiometabolic disease given their advanced age at diagnosis, high prevalence of pre-existing risk factors, and receipt of systemic therapy that targets the androgen receptor (AR). Expert panels have stressed the importance of cardiometabolic risk assessment in the clinic and proposed evaluating key risks using consensus paradigms. Yet, there is a gap in real-world evidence for implementation of comprehensive cardiometabolic care for men with prostate cancer.

**Methods:**

This is a retrospective, descriptive study of patients with prostate cancer who were referred and evaluated in the Healthy Heart Program at MD Anderson Cancer Center, which was established to mitigate cardiometabolic risks in men with prostate cancer. Patients were seen by a cardiologist and exercise physiologist to evaluate and manage cardiometabolic risk factors, including blood pressure, cholesterol, blood glucose, tobacco use, and coronary artery disease, concurrent with management of their cancer by a medical oncologist.

**Results:**

From December 2018 through October 2021, the Healthy Heart Program enrolled 55 men with prostate cancer, out of which 35 had biochemical, locoregional recurrence or distant metastases, while all received at least a single dose of a luteinizing hormone-releasing hormone (LHRH) analog. Ninety-three percent of men were overweight or obese, and 51% had an intermediate or high risk of atherosclerotic cardiovascular disease at 10 years based on the pooled cohort equation. Most men had an overlap of two or more cardiometabolic diseases (84%), and 25% had an overlap of at least 4 cardiometabolic diseases. Although uncontrolled hypertension and hyperlipidemia were common among the cohort (45% and 26%, respectively), only 29% of men followed up with the clinic.

**Conclusions:**

Men with prostate cancer have a high burden of concurrent cardiometabolic risk factors. At a tertiary cancer center, the Healthy Heart Program was implemented to address this need, yet the utility of the program was limited by poor follow-up possibly due to outside cardiometabolic care and inconvenient appointment logistics, a lack of cardiometabolic labs at the time of visits, and telemedicine visits.

## Background

Prostate cancer primarily afflicts older men with a median age at diagnosis of 67, and it has become a chronic disease due to advances in cancer care. For men with localized disease, 10-year cancer-specific survival (CSS) is 99% [1]. Median overall survival (OS) from diagnosis of metastatic prostate cancer is greater than six years in clinical trial populations in the United States [2]. Thus, men with prostate cancer are at risk for competing morbidity and mortality from age-related cardiometabolic diseases. These competing cardiometabolic diseases attenuate the benefit of cancer-centric therapeutic advances and may, in part, account for the modest improvements in OS observed at a population level [3]. The backbone of systemic treatment for advanced prostate cancer targets the androgen receptor (AR) pathway, which also adversely impacts the cardiometabolic risk factors of the patient. Oncologists utilize luteinizing hormone-releasing hormone (LHRH) analogs to suppress testicular production of testosterone and frequently intensify androgen signaling inhibition (ASI) using AR antagonists or CYP17A1 inhibition. These systemic approaches can produce cardiometabolic toxicities that include adverse body composition changes with gain in fat mass and loss of muscle mass, reduced cardiorespiratory fitness, dyslipidemia, increased insulin resistance, and ultimately cardiovascular disease [4, 5].

At the population level, atherosclerotic cardiovascular disease (ASCVD) is a major competing cause of mortality for men with localized and metastatic prostate cancer, with ASCVD defined as nonfatal myocardial infarction, death due to coronary artery disease, or stroke [6–8]. The potential risk for serious cardiometabolic toxicities in men with potentially lethal prostate cancer has been recognized for years. In 2010, the United States Food and Drug Administration (FDA) issued package inserts warnings to LHRH analogs regarding the potential risk for cardiometabolic complications. Subsequently, the American Heart Association (AHA), American Cancer Society (ACS), and American Urological Association (AUA) issued a joint statement that recommended assessment of cardiometabolic risk factors in men initiating LHRH analogs [9]. In 2016, a group of investigators published the “ABCDE” risk mitigation paradigm in an attempt to standardize cardiometabolic risk management in men receiving androgen deprivation therapy (ADT) by addressing awareness, blood pressure, cholesterol/cigarettes, diabetes, and exercise [10]. Despite multiple panel recommendations, a cross-sectional analysis of 90,494 United States veterans who received care within a single healthcare system found that only 68% received comprehensive cardiometabolic risk factor assessment and 54% had uncontrolled risk factors [11]. Thus, there is an unmet need for real-world evidence to demonstrate how to assess and manage cardiometabolic risk in men simultaneously receiving care for their prostate cancer.

Herein, we describe the clinical characteristics of prostate cancer patients referred to a cancer center-based program to manage cardiometabolic risk, the MD Anderson Healthy Heart Program, including blood pressure, cholesterol, blood glucose, tobacco use, and coronary artery disease. The challenges of implementing an ASCVD prevention program in this patient population are also described.

## Methods

This is a descriptive, retrospective study of men with prostate cancer who were referred to the Healthy Heart Program at the University of Texas MD Anderson Cancer Center (UT MDACC; Houston, TX, USA) by their treating medical oncologist. This study was approved by the Institutional Review Board of UT MDACC. Descriptive statistics were used since the study does not include formal hypothesis testing.

In 2016, UT MDACC implemented the Healthy Heart Program with the goal of mitigating cardiometabolic risk through medical management and a personalized exercise program in cancer patients and survivors. Patients were referred to the program by their medical oncologists at MDACC, but it did not include referrals from Urology or Primary Care. Patients were seen by a cardiologist to assess cardiometabolic risk factors and self-efficacy for exercise. For eligible patients evaluated in person, a cardiopulmonary exercise test (CPET) or 6-min walk test was performed to objectively measure the exercise capacity of patients. Following the completion of functional testing, the patient received counseling from the exercise physiologist on the significance of achieving the recommended exercise guidelines outlined by the American College of Sports Medicine (ACSM) to mitigate the risk of cardiometabolic disease. The ACSM guidelines recommend engaging in a minimum of 150 min of moderate-intensity physical activity or 75 min of vigorous-intensity physical activity per week. In conjunction with the counseling process, the patient and exercise physiologist collaboratively established three-month exercise goals aimed at achieving the recommended exercise guidelines. Additional follow-up was recommended based on needs and long-term goals as assessed by the cardiologist and exercise physiologist, and it was typically 3–6 months after initial consultation unless patients preferred otherwise. Communication between the cardiologists and medical oncologists was performed as needed electronically. Due to COVID-19-related precautions that began in March 2020, the Healthy Heart Program began using a telehealth model for consults and follow-ups, making exercise testing unfeasible. However, patients received counseling on the significance of adhering to the exercise guidelines recommended by the ACSM to mitigate the risk of cardiometabolic disease.

The objective of this study was to evaluate the utility of the Heart Healthy program at a tertiary cancer center to inform future implementation at academic centers in the United States. Thus, we describe baseline cancer and cardiometabolic characteristics of men with prostate cancer referred to the Heart Healthy Program. Global cardiometabolic health was measured using the American College of Cardiology 10-year ASCVD risk estimator (https://tools.acc.org/ascvd-risk-estimator-plus/#!/calculate/estimate/), and patients were considered unevaluable if blood pressure or cholesterol readings were unavailable at time of visit. From the estimator, the 10-year risk for ASCVD is categorized as: low risk (< 5%), borderline risk (5 – 7.4%), intermediate risk (7.5 – 19.9%), or high risk (≥ 20%). Uncontrolled hypertension was defined as a systolic blood pressure ≥ 140 mm Hg or diastolic blood pressure ≥ 90 mm Hg, and uncontrolled hyperlipidemia was defined as a LDL ≥ 130 mg/dL or total cholesterol ≥ 240 mg/dL. These thresholds were selected to align with the large Veterans Affairs analysis of cardiometabolic risk factors in men with prostate cancer as well as other large retrospective experiences [11, 12]. Then, we report how the Heart Healthy impacted cardiometabolic health in patients from this cohort with at least one follow-up visit.

## Results

Between December 2018 and October 2021, 55 men with prostate cancer were referred to the Healthy Heart Program to optimize and manage cardiometabolic health. Of these men, 35 had advanced disease and 20 had localized prostate adenocarcinoma. Among those with advanced disease, 16 had biochemical or locally recurrent disease, 14 had metastatic hormone sensitive prostate cancer (mHSPC), and 5 had metastatic castration-resistant prostate cancer (mCRPC). Most of the men were receiving systemic therapy at the time of their initial Healthy Heart visit (82%, *n* = 29), and their median prostate-specific antigen (PSA) was 0.1 ng/dL (interquartile range, IQR, 0—5.5). The most used systemic therapy was LHRH agonist or antagonist alone (52%, *n* = 15) followed by LHRH agonist/antagonist plus ASI (45%, n = 13) and one patient received cytotoxic chemotherapy (3%). The median time from initiation of systemic therapy to clinic visit was 150 days (IQR 66 – 798 days).

Median age of our cohort was 67 years at time of clinic visit (IQR 64 – 71.5 years), and almost all men were overweight or obese (93%, Table 1). For men evaluated in person (*n* = 49), median systolic blood pressure (SBP) was 137 mmHg [standard deviation (SD) = 16.4] and diastolic blood pressure (DBP) was 77 mmHg (SD = 9.8). Median low-density lipoprotein (LDL) level was 124 and triglyceride level was 159 as shown in Table 1 (*n* = 31), and median hemoglobin A1C was 5.9% (*n* = 29). For evaluable men (*n* = 30), 26% had a high 10-year ASCVD risk (≥ 20%) and 26% had an intermediate risk (7.5 – 20%) based on the pooled cohort equation. The frequency of cardiometabolic disease at initial visit is shown in Table 2. Fifteen percent of men had a prior history of coronary artery disease, while prior cerebrovascular accident was rare (1.8%). Most men had two or more cardiometabolic diseases, defined as diabetes mellitus, hypertension, hyperlipidemia, obesity, or coronary artery disease, (84%) with hyperlipidemia and hypertension being the most prevalent at 69%, respectively, and 26% men had an overlap of at least 4 cardiometabolic diseases.

**Table 1:** Baseline objective cardiometabolic measures in men seen in the Heart Healthy clinic

	**All** (*n* = 55)
Age at visit (Range)	67 (44, 82)
Total cholesterol (Median, IQR)	212 (176, 243.8)
Low-density lipoprotein (*n* = 31, median, IQR)	123.5 (97, 168)
High-density lipoprotein (Median, IQR)	52 (43.5, 59.3)
Triglycerides (*n* = 31, median, IQR)	159 (111, 205.3)
Hemoglobin A1C (*n* = 29, median, IQR)	5.9 (5.6, 6.2)
Body mass index	
Normal	4 (7.3%)
Overweight	18 (32.7%)
Obese	33 (60%)
Blood pressure at visit (*n* = 49, median)	
Systolic	137
Diastolic	77
ASCVD risk score	
< 5%	1 (1.8%)
5—7.5%	1 (1.8%)
7.5–20%	14 (25.5%)
> 20%	14 (25.5%)
NA	25 (45.4%)
Subsequent follow up	
Yes	16 (29.1%)
No	39 (70.9%)

**Table 2:** Prevalence of cardiometabolic comorbidities on initial evaluation

**Cardiometabolic comorbidity**	**Prevalence in cohort** (*n* = 55)
Coronary artery disease	
Yes	8 (14.6%)
No	47 (85.4%)
Prior cerebrovascular accident	
Yes	1 (1.8%)
No	54 (98.2%)
Hyperlipidemia	
Yes	38 (69.1%)
No	17 (30.9%)
Hypertension	
Yes	38 (69.1%)
No	17 (30.9%)
Tobacco abuse	
Current smoker	1 (1.8%)
Former smoker	18 (32.7%)
Non-smoker	36 (65.5%)
Diabetes mellitus	
Yes	14 (25.5%)
No	41 (74.5%)
Comorbidity overlap	
1 comorbidity	9 (16.4%)
2 comorbidities	17 (30.9%)
3 comorbidities	11 (20%)
≥ 4 comorbidities	14 (25.5%)

At initial visit, 60% of men were taking a statin, 47% an anti-platelet agent, and 64% one or more anti-hypertensive agents (Table 3). However, men frequently presented with uncontrolled hypertension (45%, defined as either SBP > 140 mmHg or DBP > 90 mmHg) and and/or hyperlipidemia (26%, defined as LDL ≥ 130 mg/dL or total cholesterol ≥ 240 mg/dL). After the cardiologist provided cardiometabolic risk reduction counseling and a personalized exercise program was developed, only 29% (*n* = 16) of men engaged in follow-up exercise counseling and/or clinic reassessment. Among these 16 men, median time to follow-up appointment was 185 days (IQR 160—259). Of men with weight recorded at baseline and follow-up, 58.3% (*n* = 7/12) gained weight with median increase of 2.8 kg. Of men with lipid panels at both time points, 66.7% (*n* = 6/9) had a decrease in total cholesterol (median is -57) and in LDL (median is -52). As a sum measure of cardiometabolic risk mitigation, the 10-year ASCVD decreased in 3/7 men with available data. The ASCVD risk score could not be calculated at follow-up in eight men due to missing data, and 6/8 of these men had telemedicine visits for follow-up due to the COVID-19 pandemic.

**Table 3:** Utilization of medications to modify cardiometabolic health among men seen in the Heart Healthy Clinic

**Use of cardiometabolic mediation at visit**	**Prevalence in cohort** (*n* = 55)
Statin	
Yes	33 (60%)
No	22 (40%)
Anti-platelet therapy	
Yes	26 (47.3%)
No	29 (52.7%)
Anti-hypertensive	
Yes	35 (63.6%)
No	20 (36.4%)
Beta blocker	
Yes	16 (29.1%)
No	39 (70.9%)
Metformin	
Yes	8 (14.5%)
No	47 (85.5%)
Other diabetic medication	
Yes	10 (18.2%)
No	45 (81.8%)

A total of 7 patients completed CPET on the treadmill, and the mean VO_2peak_ was 22.2 ± 6.5 (mL/kg/min), which is the equivalent of 24.5% below healthy, age-adjusted reference values. Six patients performed the 6-min walk test, and the mean distance was 389.1 ± 63.4 m, the equivalent of 21.5% below healthy, age-adjusted reference values.

## Discussion

Prostate cancer is unique among malignancies as advances in cancer therapy have made it a chronic disease in an older population who often have age-related medical comorbidities. The Healthy Heart Program was established at MD Anderson Cancer Center with the goal of providing comprehensive cardiometabolic heath assessment and management by expert cardiologists and exercise physiologists to compliment the cancer-centric care provided by medical oncologists. In our experience, men with prostate cancer referred to the Heart Healthy clinic had a high burden of cardiometabolic risk factors with 93% being overweight or obese, 51% having intermediate or high-risk for ASCVD within the next 10 years, and 84% having at least two cardiometabolic diseases. Like the United States Veterans Affairs (VA) hospital experience, many men had uncontrolled cardiometabolic disease [13]. However, patients seen in the Healthy Heart Program often did not follow up, which attenuated the impact of the clinic on men’s cardiometabolic health, despite buy-in from referring oncologists, the institution, and cardiologists. This experience highlights the need to move from expert panels and recommendations to real-world data that demonstrates how to effectively implement cardiometabolic care for men with prostate cancer and reinforce the value to patients, oncologists, and institutions. Furthermore, it is critical that we elucidate the shared biology underlying differential cardiometabolic disease overlap in men with prostate cancer [14].

This study demonstrates the challenges we encountered implementing the Healthy Heart Program at a tertiary cancer center. Objectively, barriers to implementation were men not having cardiometabolic labs at the time of their Healthy Heart visit (45%), poor follow-up and compliance (71%), and telemedicine visits adversely impacting the ability of our cardiologists to assess the effectiveness of their recommendations. Anecdotally, our group also encountered challenges with patients already being established with local cardiologists and intermittent, in person follow-up due to our national patient population as a tertiary cancer center. The 55 men evaluated in the Heart Healthy program also represents a small fraction of the total number of patients with prostate cancer seen at our center. In our experience, a subset of the medical oncologists referred patients to the Heart Healthy program, and there appeared to be a bias towards referring patients with more pressing cardiometabolic risk factors. This experience raises the question of how cardiometabolic care should be delivered for men with prostate cancer who receive care at a referral cancer center. There are benefits to having coordinated care between medical oncologists who prioritize global patient health and cardiologists at a cancer center with experience with side effects of hormonal therapies. We observed this as patients who followed up had decreases in lipid levels and some had decreases in 10-year ASCVD risk with short interval follow-up. This coordinated care could become more efficient with a comprehensive prostate cancer care clinic where medical oncology, cardiology, and other toxicity-related care providers, such as psychology and urologic men’s health, work in the same proximity to eliminate the risk of missing labs, telemedicine visits, and poor follow-up. Alternatively, the comprehensive cardiometabolic care that can be provided by local cardiologists and internists may be preferable for patients with complex cardiometabolic disease or limited interest in long-term follow-up. For these patients, the need for interhospital dialogue is critical. Looking forward, a potential solution for this dilemma encountered at tertiary cancer centers is to utilize the expertise of cardio-oncologists and medical oncologists to identify risk factors during consultation and provide recommendations for cardiometabolic toxicity mitigating strategies to local oncologists, cardiologists, and internists who can more effectively implement and follow-up the changes. Additionally, tobacco cessation is among the most challenging cardiometabolic risk factors to mitigate clinically, so there is potential for coordinated cardiometabolic risk mitigation to meaningfully lower events since only 1.8% of patients in our cohort actively used tobacco.

Over the past decade, the United States FDA, professional societies, and expert panels have stressed the importance of cardiometabolic health for men with prostate cancer, but our challenges with implementation are not unique as a multicenter clinical trial and a cross-sectional analysis of the United States VA hospital have made similar observations [11, 15]. Across all settings, a challenge in implementation is the shift from cancer being the dominant focus of a patient’s medical care to a chronic disease managed over years to decades. Prostate cancer is a contemporary model for this transition, yet therapeutic advances will make this a common issue across oncology. A cancer diagnosis is an impactful moment in anyone’s life journey, and cancer becomes an active problem that the patient and clinician prioritize and treat aggressively. In contrast, cardiometabolic diseases are chronic, impacted by lifestyle, and at risks for subsequent major adverse cardiovascular events (MACE). In our current paradigm, cardiometabolic health represents preventative medicine that competes for time and financial resources with an active health problem, cancer. As clinical investigators and clinicians, we must shift the cognitive framework for prostate cancer in a subset of men to define prostate cancer as an age-related event that shares biology with other cardiometabolic diseases [14]. Thus, caring for cardiometabolic health also becomes caring for the prostate cancer. This aligns with the definition of prostate cancer survivorship proposed by the SuRECap working group as encompassing the physical, psychological, and societal effects of prostate cancer therapy, from time of diagnosis through remainder of life [16]. While the importance of this framework shift is clear, clinicians are also challenged by the nuances and evolution of cardiometabolic risk factor mitigation. For example, we defined uncontrolled hyperlipidemia as an LDL ≥ 130 mg/dL or total cholesterol ≥ 240 mg/dL to align with prior efforts; however, the 2018 American College of Cardiology (ACC) guidelines recommend statin therapy for any patient 40 to 75 years of age with an LDL ≥ 70 mg/dL and a 10-year ASCVD risk score ≥ 7.5% [17]. Ultimately, the optimal lipid and blood pressure goals men with prostate cancer is unknown and complicated by their older age and risk for competing mortality, which affects 10-year risks to benefit ratios.

Due to substantial gaps in prostate cancer survivorship research, the SuRECaP working group recommended research to address three key items: the biology of treatment toxicity, the clinical effects of therapy, and patient-reported outcomes with therapy [16]. The PRONOUNCE trial aimed to investigate clinical effects of LHRH analogs on the relative risk of MACE, yet the trial was terminated early due to slow accrual and a lower incidence of MACE than planned [18]. There are multi-center clinical trials currently investigating the risk of cardiometabolic disease in men with prostate cancer and its relationship to androgen-deprivation therapy (RADICAL PC; NCT03127631) and the impact of high intensity aerobic and resistance exercise on overall survival in men with metastatic prostate cancer (INTERVAL-GAP4; NCT02730338) [19, 20]. The challenges we encountered implementing the Healthy Heart Program informed our current research for cardiometabolic health and host toxicity with ASI. Currently, we are conducting a randomized, phase II clinical trial to test the hypothesis that a 16-week digital risk factor modification program will improve 10-year ASCVD risk score as compared to usual care in men with potentially lethal prostate cancer receiving at least LHRH agonist or antagonist (ProTrio; NCT05054296). This study will also facilitate translational research to optimize the therapeutic index with ASI by accounting for the biology underlying host toxicity with ASI.

## Conclusions

Men with prostate cancer have a high burden of concurrent cardiometabolic risk factors. At a tertiary cancer center, the Healthy Heart Program to address this need was limited by poor follow up, despite many men having uncontrolled cardiometabolic disease. This experience highlights the need for real-world data that demonstrates how to effectively implement cardiometabolic care for men with prostate cancer.

## Data Availability

The datasets used and/or analyzed during the current study are available from the corresponding author on reasonable request.
